# Shading and Watering as a Tool to Mitigate the Impacts of Climate Change in Sea Turtle Nests

**DOI:** 10.1371/journal.pone.0129528

**Published:** 2015-06-01

**Authors:** Jacob E. Hill, Frank V. Paladino, James R. Spotila, Pilar Santidrián Tomillo

**Affiliations:** 1 Department of Biology, College of Arts and Sciences, Indiana University-Purdue University Fort Wayne, Fort Wayne, Indiana, United States of America; 2 Department of Biodiversity, Earth and Environmental Science, Drexel University, Philadelphia, Pennsylvania, United States of America; 3 Population Ecology Group, Institut Mediterrani d’ Estudis Avançats, IMEDEA (CSIC-UIB), Mallorca, Spain; Deakin University, AUSTRALIA

## Abstract

Increasing sand temperatures resulting from climate change may negatively impact sea turtle nests by altering sex ratios and decreasing reproductive output. We analyzed the effect of nest shading and watering on sand temperatures as climate mitigation strategies in a beach hatchery at Playa Grande, Costa Rica. We set up plots and placed thermocouples at depths of 45cm and 75cm. Half of the plots were shaded and half were exposed to the sun. Within these exposure treatments, we applied three watering treatments over one month, replicating local climatic conditions experienced in this area. We also examined gravimetric water content of sand by collecting sand samples the day before watering began, the day after watering was complete, and one month after completion. Shading had the largest impact on sand temperature, followed by watering and depth. All watering treatments lowered sand temperature, but the effect varied with depth. Temperatures in plots that received water returned to control levels within 10 days after watering stopped. Water content increased at both depths in the two highest water treatments, and 30 days after the end of water application remained higher than plots with low water. While the impacts of watering on sand temperature dissipate rapidly after the end of application, the impacts on water content are much more lasting. Although less effective at lowering sand temperatures than shading, watering may benefit sea turtle clutches by offsetting negative impacts of low levels of rain in particularly dry areas. Prior to implementing such strategies, the natural conditions at the location of interest (e.g. clutch depth, environmental conditions, and beach characteristics) and natural hatchling sex ratios should be taken into consideration. These results provide insight into the effectiveness of nest shading and watering as climate mitigation techniques and illustrate important points of consideration in the crafting of such strategies.

## Introduction

All species of sea turtles exhibit an oviparous reproductive strategy that requires gravid females to return to their natal beaches to lay eggs. While eggs remain buried in the ground during the 45–65 day incubation period, they are exposed to an array of environmental variables that influence the development of eggs and hatchlings [1–3]. During this time, developing embryos are also impacted by alterations to the nest environment induced by the eggs themselves [4,5].

Incubation temperatures affect sex ratios of hatchlings [6], duration of the incubation period [3,7], and hatching and emergence success [8]. There can also be long-term impacts from incubation temperatures because they impact the operational sex ratios of adults [9] and population dynamics [10]. Sea turtles have temperature-dependent sex determination, with warmer nests producing female hatchlings and cooler nests producing males [11,12]. The temperature of the nest is influenced by physical traits of the beach, such as sand albedo [13] and vegetation cover [6], but is mainly impacted by prevailing climatic conditions. High levels of precipitation can reduce nest temperature and increase production of male hatchlings [3,14]. Seasonal changes in air temperature can cause shifts in hatchling sex ratios over the course of the nesting season [3,15] and may also drive long-term changes in incubation conditions [16].

The range of incubation temperatures that produces both sexes is called the transitional range (TR) and typically only spans 1–4°C [17]. In leatherback turtles (*Dermochelys coriacea*), the TR is 1°C or less [11,18]. The small TRs found in sea turtles means that even small changes in temperature can have significant consequences for hatchling sex ratios.

Temperature during incubation also influences hatching success. In olive ridley turtles (*Lepidochelys olivacea*), incubation temperatures greater than 35°C result in the death of developing embryos and failure to produce any hatchlings [19]. High temperatures can also be fatal to hatchlings during the pre-emergence period, causing hatchlings to die at the surface of the nest after hatching, which can also greatly decrease hatchling production [7,20].

Temperature also interacts with water content of the sand to influence hatchling morphology in turtles. Shorter incubation times experienced by warmer nests can result in less yolk being converted to tissue [21], which results in the production of hatchlings that are smaller [22,23] but have higher energy stores. Smaller turtle hatchlings are less adept at terrestrial motion [24], crawl slower, and swim slower [23], but may survive better in energy-poor environments [25]. However, larger hatchlings are less available to gape-limited predators. Furthermore, their faster swimming ability likely enables them to more successfully navigate through the large aggregations of predators offshore from the nesting beach [25]. Assuming hatchlings have access to adequate food resources upon entering the water, the decreased size and speed of hatchlings from warmer and drier nests likely results in lower survival rates.

The influence of environmental conditions on egg development means that sea turtle nests are subject to stochastic environmental events and climate change has the potential to dramatically alter incubation conditions [26–29]. Central America is predicted to be more impacted by climate change than any other region of the tropics [30]. The Intergovernmental Panel on Climate Change (IPCC) projections showed that Central America will warm by 2–3°C over the next century [31]. Combined with the expected decreases in precipitation, the region will become increasingly hotter and drier in the coming decades.

In the Eastern Pacific, Playa Grande, Costa Rica is one of the largest remaining leatherback nesting beaches. At this site, low levels of precipitation and high temperatures have been linked to decreased hatching success and emergence of hatchlings from the nest [32]. Consequently, these climate driven variabilities significantly influence annual reproductive output. Years that have higher temperatures and lower levels of precipitation produce fewer hatchlings [8,32]. As Central America becomes warmer and drier throughout the 21^st^ century, increasing egg and hatchling mortality will threaten the survival of leatherback turtles in the Eastern Pacific [32,33]. Even with removal of the largest human-induced sources of mortality, the impacts of climate change alone are severe enough to drive the population toward extinction [33]. The threat to these turtles posed by impending climate change warrants an investigation into possible mitigation measures to avert its most damaging impacts.

Nest irrigation and shading have been proposed as possible climate mitigation measures for sea turtle nests [34]. In natural nests, shading produced by vegetation lowers incubation temperatures and results in the production of higher proportions of male hatchlings [6]. Shading by planting trees lowers nest temperatures and produces hatchlings with higher locomotion performance than nests that are not shaded [35]. For some populations, hatchling sex ratios could reach 100% female by 2070 [36] and shading of leatherback nests has been shown to increase male production without compromising hatchling fitness or hatching success [37]. Likewise, experimental watering under certain conditions can lower nest temperatures [38] in the same manner that rainfall can lower incubation temperatures, resulting in the production of males [14,15].

In this study, we combined shading and watering strategies to see how they interacted and influenced temperature and water content of sand at nest depth. In particular, our aims were to (1) compare the effectiveness of shading and watering in lowering sand temperatures, (2) examine the impacts of different water amounts on sand temperature and water content, and (3) compare how sand at depths of olive ridley and leatherback turtle nests responded to experimental treatments. The overall aim of this study was to provide a foundation on which to formulate future climate mitigation techniques for sea turtle nests.

## Materials and Methods

We carried out the experiment in a beach hatchery at Playa Grande (10°20’ N, 85°51’ W), Costa Rica. We set up 18 one m^2^ plots and placed a Cu-Cn thermocouple (± 0.1°C) at both 45cm and 75cm depths in the center of each plot. These depths corresponded to the respective mean bottom nest depths of olive ridley and leatherback turtles, the two species that nest in the area. We completely shaded nine plots with fence mesh and left nine plots exposed to the sun. Within each of the shade/exposure treatments, we applied three watering treatments based on the highest (721 mm), lowest (100 mm), and average (323 mm) amount of rainfall registered in North Pacific Costa Rica in October between 1976 and 2009.

October is the rainiest and the last month of the rainy season in North Pacific Costa Rica. It also coincides with the beginning of the nesting season for leatherback turtles (October-March) [8] and the middle of the season for olive ridley turtles (August-January) [39]. Precipitation accumulated during this month influences hatching success and emergence rates of leatherback hatchlings [32]. November is a transitional month and has low levels of precipitation and the dry season (December-April) has very little or no precipitation. Climatic conditions are highly variable in this area and are affected by El Niño Southern Oscillation (ENSO). Patterns of rainfall in the Eastern Pacific associated with ENSO vary significantly across small spatial scales [40]. In Northwest Costa Rica, years with La Niña conditions result in high levels of precipitation, while years with El Niño are dry and frequently result in droughts [40]. The amounts of water used in this study corresponded to La Niña, El Niño, and neutral conditions, respectively [32].

We did three replicates for each treatment to account for spatial variability within the hatchery. Replicates were randomly distributed throughout the hatchery and each experimental plot was surrounded by empty plots to ensure that they were not influenced by water applied to adjacent plots. Space in the hatchery was limited. We chose the number of replicates per experimental treatment based on the maximum number of plots that could be arranged in the hatchery while still allowing these buffer plots and providing space for clutches relocated from the beach that were incubating. Carrying out this experiment during the nesting season limited space in the hatchery due to incubating clutches, but the nesting season encompasses most of the dry season. It was important to conduct the study during this time to ensure that rainfall did not add additional water to plots and to have proper controls that received no water.

We watered plots daily in the afternoon and applied water in equal amounts per day over the course of 31 days. Temperatures were recorded every other day in the afternoon (15:00–16:00) using a Bat 12 (±0.1°C) or HH200A Omega (±0.1°C) Handheld Thermometer thermocouple reader. After we completed the water treatment, we continued taking temperatures through the following month, maintaining the shaded structure during this time. We also monitored temperatures using four HOBO temperature loggers (±0.53°C, Onset Computer Corporation, Bourne MA) to record temperatures hourly throughout the experiment. We placed these loggers at 45cm and 75cm in an exposed and shaded plot that received the high water treatment. These devices were used to measure daily fluctuations in temperature that may not have been apparent with thermocouple measurements alone, which were only taken once per day. A weather station near the hatchery recorded air temperatures.

To measure the water content of the sand, we took sand samples from each plot at depths of 45cm and 75cm (1) before treatments started, (2) the day after treatments were completed, and (3) 30 days after the end of the watering experiment. We dug a hole in the plots, used a meter stick to determine the depth and collected samples at depth from the edge of the cavity using a shovel. We weighed 20 g from each sand sample and immediately dried the sample in an oven at 100°C for 48 hours. We previously determined that mass of the samples remained constant after 48 hours in the oven. After that time, we weighed the samples again to determine their dry mass. We used wet mass (*M*_*w*_) and dry mass (*M*_*d*_) to calculate the gravimetric water content (*W*) using the equation W=(Mw−Md)/Md. Field work took place at Parque Nacional Marino Las Baulas under permits from the Ministerio de Ambiente, Energía y Telecomunicaciones de Costa Rica (MINAET).

We performed a general linear univariate model to examine the effects of depth, shade treatment, and water treatment on temperature, as well as possible interactions between these variables. Subsequently, we used post-hoc testing with Bonferroni corrections to examine within group differences between treatments. These tests were applied on the temperatures recorded (1) during the month of water treatment and (2) during the one month after the water treatment was completed. We used SPSS statistics v. 20.0 [41] for all statistical analyses.

## Results

### Effects on temperature

Depth, amount of water received, and shade significantly influenced temperature during the course of treatments (p < 0.001 all cases). Shading had the largest impact (Ƞ^2^ = 0.566), followed by water treatment (Ƞ^2^ = 0.441) and depth (Ƞ^2^ = 0.138). There were significant interactions between depth and shade treatment (p < 0.001) and between depth and water treatment (p < 0.001), but not between shade and water treatment (p = 0.360). The Bonferroni post hoc test showed significant differences in temperature between the control and all water treatments (p < 0.001 in all cases), between low and average treatments (p < 0.01) and between low and high treatments (p = 0.001), but not between average and high treatments (p > 0.05).

**Fig 1:**
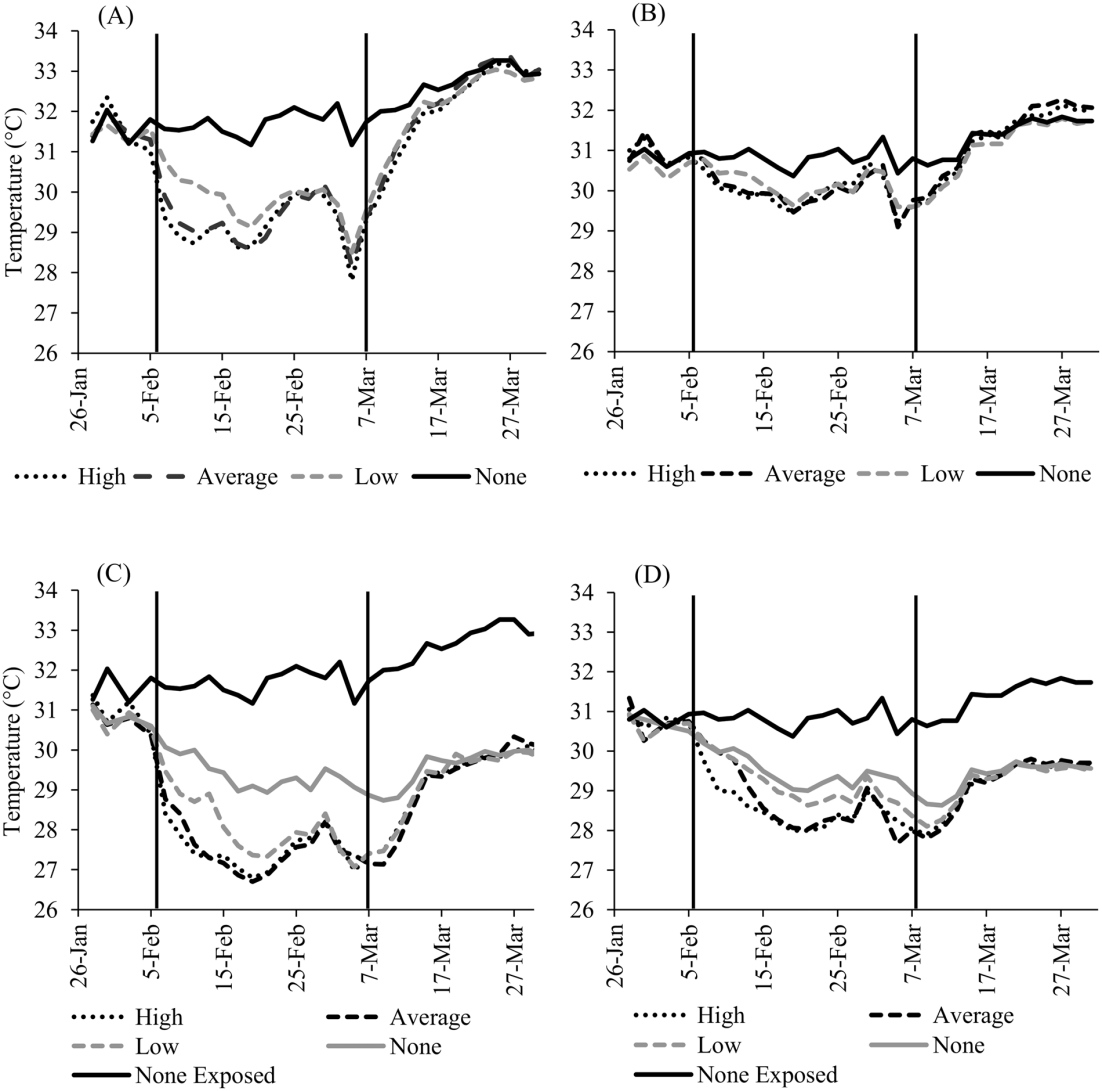
Sand temperature (°C) through time at olive ridley (A,C) and leatherback (B,D) turtle nest depths. (A) 45cm exposed. (B) 75cm exposed. (C) 45cm shaded. (D) 75cm shaded. Water treatments were: high (721 mm), average (323 mm) and low (100 mm), corresponding to La Niña, neutral and El Niño conditions in Northwest Costa Rica. Vertical lines mark the beginning and end of watering treatments.

When looking at depths separately, the Bonferroni post hoc test showed that there were significant differences in sand temperature at 45cm between control and all water treatments (p < 0.001) and between low and high treatments (p < 0.05), but not between low and average (p = 0.06) and average and high treatments (p = 1.00). During the month of watering, in exposed plots at 45cm, temperatures in the low water treatment were on average 1.8°C lower than the controls, while average and high treatments were 2.3°C and 2.4°C lower, respectively (Fig 1A, Table 1). Temperatures in shaded plots with no water averaged 2.2°C lower than exposed plots with no water, while low water treatments were 3.5°C lower, and both average and high treatments were 4.0°C lower. Each of the shaded plots with water treatments were 1.7°C lower than those in exposed plots with the same water treatment (Fig 1C).

**Table 1:** Mean sand temperatures (°C) (±SD) at 45cm depth for each experimental treatment.

Shade Treatment	Exposed Plots				Shaded Plots			
Water Treatment	None (0 mm)	Low (100 mm)	Average (323 mm)	High (721mm)	None (0 mm)	Low (100 mm)	Average (323 mm)	High (721mm)
Temperature during 31 days of water application	31.7±0.3	29.9±0.7	29.4±0.7	29.3±0.7	29.5±0.5	28.2±0.9	27.7±0.9	27.7±0.9
Temperature during 30 days following water application	32.6±0.5	32.0±1.0	32.1±1.2	32.0±1.2	29.6±0.5	29.2±0.9	29.1±1.1	29.2±1.0

Temperatures were recorded during the 31 days of water application and for 30 days after water treatments stopped. Water treatments were: high (721 mm), average (323 mm) and low (100 mm), corresponding to La Niña, neutral and El Niño conditions in Northwest Costa Rica.

At 75cm, there were significant differences in temperature between control and all water treatments (p < 0.05 all cases), but not among the treatments (p > 0.05). In exposed plots at 75cm, temperatures in the low water treatment averaged 0.6°C lower than in plots with no water and temperatures in average and high treatments were 0.7°C lower (Fig 1B, Table 2). Temperatures in shaded plots with no water averaged 1.3°C lower than temperatures in exposed plots without water. Low, average, and high water treatments in shaded plots were respectively 0.9°C, 1.2°C, and 1.4°C lower than temperatures in the same water treatment in exposed plots (Fig 1D).

**Table 2:** Mean sand temperatures (°C) (±SD) at 75cm depth for each experimental treatment.

Shade Treatment	Exposed Plots				Shaded Plots			
Water Treatment	None (0 mm)	Low (100 mm)	Average (323 mm)	High (721mm)	None (0 mm)	Low (100 mm)	Average (323 mm)	High (721mm)
Temperature during 31 days of water application	30.8±0.2	30.2±0.4	30.1±0.5	30.1±0.4	29.5±0.4	29.3±0.6	28.9±0.9	28.7±0.7
Temperature during 30 days following water application	31.4±0.4	31.0±0.8	31.3±0.9	31.2±0.9	29.3±0.4	29.1±0.5	29.1±0.7	29.1±0.7

Temperatures were recorded during the 31 days of water application and for 30 days after water treatments stopped. Water treatments were: high (721 mm), average (323 mm) and low (100 mm), corresponding to La Niña, neutral and El Niño conditions in Northwest Costa Rica.

Temperatures in watered plots returned to control levels within 10 days after the end of watering. There was not a significant effect of water treatment on temperature during the month after watering was completed. Depth affected temperature significantly (p < 0.001), but to a much lesser degree than during the watering (Ƞ^2^ = 0.088), and shade continued to exert a strong influence on temperature (Ƞ^2^ = 0.689, p < 0.001). During this time, at both depths none of the water treatments were statistically different from plots that did not receive water (p > 0.05 all cases).

**Fig 2:**
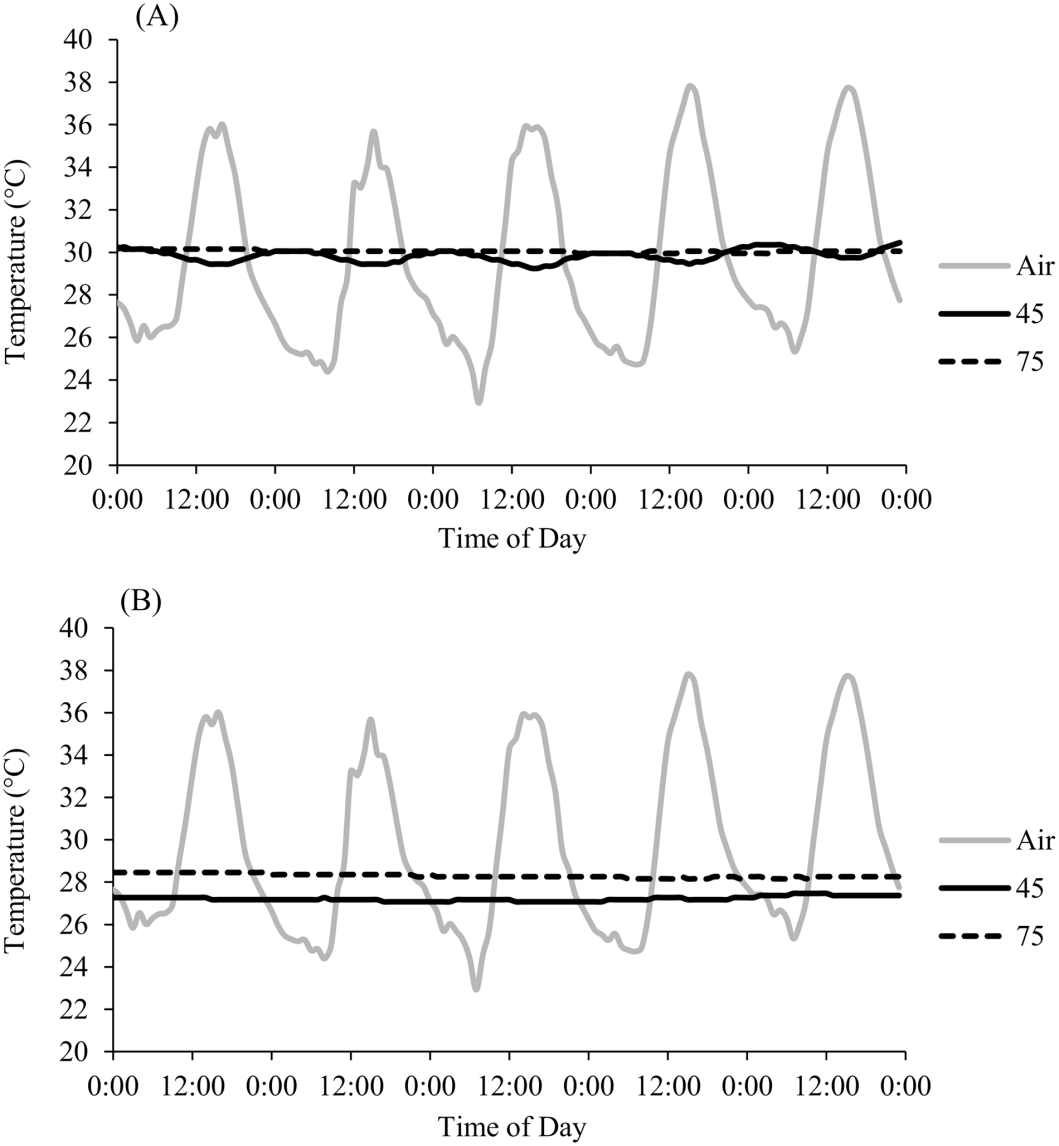
Air and sand temperatures (°C) at 45 and 75 cm depth from data loggers. Temperatures are shown over a five day period during watering from (A) exposed and (B) shaded plots that received high water treatments (721 mm).

There was little fluctuation in sand temperature in experimental plots, as indicated by small standard deviations in HOBO temperature measurements. Average temperatures recorded in the exposed plots were 29.84 ± 0.28°C and 30.05 ± 0.06°C at 45cm and 75cm, respectively (Fig 2A). In the shaded plots, respective average temperatures were 27.21 ± 0.11°C and 28.30 ± 0.10°C at 45cm and 75cm (Fig 2B). By contrast, average air temperature across the same time period was 29.65 ± 4.07°C.

### Effects on Water Content

After the month of watering, water content decreased in controls, showed little change in low water treatments, and increased in both average and high water treatments. At 45cm, the average initial water content was 0.037 ± 0.02 g g^-1^. After the month of watering, water content in the control exposed plot fell to 0.013 g g^-1^ because of lack of rain and increase in air temperatures, and decreased slightly in low exposed and low shaded treatments, dropping to 0.030 and 0.028, g g^-1^ respectively (Table 3). In the remaining plots, water content increased to 0.058, 0.069, 0.070, and 0.078 g g^-1^ in the average shaded, average exposed, high shaded, and high exposed plots, respectively.

**Table 3:** Mean gravimetric water content (g g^-1^) in experimental plots at 45 cm depth.

Shade Treatment	Exposed Plots				Shaded Plots			
Water Treatment	None (0 mm)	Low (100 mm)	Average (323 mm)	High (721mm)	None (0 mm)	Low (100 mm)	Average (323 mm)	High (721mm)
Water content (g g^-1^) at end of water application	0.013	0.030	0.069	0.078	-	0.028	0.058	0.070
Number of Samples	1	2	3	3	0	3	3	3
Water content (g g^-1^) at 30 days after end of water application	-	0.021	0.036	0.049	0.018	0.028	0.030	0.033
Number of Samples	0	3	3	3	1	3	3	3

Treatments marked with (-) could not be sampled because sand was too dry and collapsed before the target depth could be reached. Differences in number of samples per treatment also reflect difficulties in sampling from some plots because of dry sand. Water treatments were: high (721 mm), average (323 mm) and low (100 mm), corresponding to La Niña, neutral and El Niño conditions in Northwest Costa Rica.

Between the end of watering and the following month, water content remained the same in the low shaded treatment but decreased in all other plots to 0.021, 0.036, 0.030, 0.049, and 0.033 g g^-1^ in the low exposed, average exposed, average shaded, high exposed, and high shaded plots, respectively. Samples from the control plots could not be collected in the month after water because the upper layers of sand down to 25 cm deep were dry and the hole collapsed. Digging deeper would have mixed the dry sand with the sand below, making it impossible to collect an uncontaminated sample. In month after water application ended, among plots that were shaded, the high water treatment plots still had sand with a water content that was nearly twice that in the plots without water. Among exposed plots, water content was twice as high in the high water treatment plot as in the low water treatment plot one month after water treatments stopped (Table 3).

The same general trends were evident at 75cm, but values tended to be higher than at 45cm. The initial water content in these plots was 0.040 ± 0.17 g g^-1^. When water treatment stopped, water content in the low exposed and low shaded plots fell to 0.032 and 0.025 g g^-1^, respectively (Table 4). In the remaining plots, water content increased to 0.080, 0.066, 0.085, and 0.074 g g^-1^ in the average exposed, average shaded, high exposed, and high shaded plots, respectively. Between the end of watering and the following month, water content remained the same in the low shaded plots and decreased to 0.028, 0.055, 0.030, 0.051, and 0.038 g g^-1^ in the low exposed, average exposed, average shaded, high exposed and high shaded plots, respectively. One month after watering stopped, in exposed plots, those with high water amounts had nearly double the water content of those with low water treatments.

**Table 4:** Mean gravimetric water content (g g^-1^) in experimental plots at 75 cm depth.

Shade Treatment	Exposed Plots				Shaded Plots			
Water Treatment	None (0 mm)	Low (100 mm)	Average (323 mm)	High (721mm)	None (0 mm)	Low (100 mm)	Average (323 mm)	High (721mm)
Water content (g g^-1^) at end of water application	-	0.032	0.080	0.085	-	0.025	0.066	0.074
Number of Samples	0	1	3	3	0	3	3	3
Water content (g g^-1^) at 30 days after end of water application	-	0.028	0.055	0.051	-	0.025	0.030	0.038
Number of Samples	0	1	2	2	0	3	3	3

Treatments marked with (-) could not be sampled because sand was too dry and collapsed before the target depth could be reached. Differences in number of samples per treatment also reflect difficulties in sampling from some plots because of dry sand. Water treatments were: high (721 mm), average (323 mm) and low (100 mm), corresponding to La Niña, neutral and El Niño conditions in Northwest Costa Rica.

## Discussion

All experimental treatments significantly lowered sand temperature when compared to controls, but shading was more effective than watering. Temperatures in plots that received the highest amount of water and no shade were not significantly different from plots that were shaded with no water. Applying large amounts of water was no more effective at reducing sand temperature than just shading plots. Within water treatments, the highest amounts of water did not result in the lowest sand temperatures, indicating a threshold at which additional water does not further decrease sand temperature. Temperatures in every water treatment rebounded to control levels within 10 days after watering stopped, indicating that watering regimens must be maintained to achieve sustained reductions in temperature.

The reason for the lack of a long-term watering effect on sand temperature was that solar radiation drives the temperature of the beach. When we stopped watering, the sun heated the beach and dried out the surface layer of the sand. The effect of watering was similar to that of a storm, in which a large amount of rain falls on the beach and then the beach heats up again after the weather clears. We observed this on Playa Grande with Hurricane Michelle in 2001 [4] and at Rancho Nuevo, Mexico in 1982 [3]. Similarly, on beaches that have distinct rainy and dry seasons, nests heat up rapidly when the rainy season ends [7]. When it stops raining, even if water content does not change, solar heating heats up the beach and the upper layers of the sand dry out. Evaporation decreases to near zero and the added water no longer cools the sand, but conducts heat downward, which is what we observed in this experiment.

Although water treatments were less effective than shading at lowering temperature, they maintained or increased water content of the sand, which is important for optimal incubation of sea turtle nests. The long-term impact of water content confirms previous results from in situ leatherback nests at Playa Grande. Playa Grande is in a dry area that receives no rain during 4–5 months of the nesting season and water received in the two months prior to nest deposition is one of the greatest factors influencing hatching success, even more important than ambient temperatures [32]. Rainfall is a better predictor of hatching success of leatherback eggs at Playa Grande than air temperature because water content of the beach at nest depth is maintained through time, and temperatures at nest depth are not too high for egg development. Watering of nests at the beginning of the season may serve to offset the decrease in precipitation during this time of the year that is predicted in the region under future climate change scenarios [33].

Watering at the beginning of the season would also help to offset the negative impacts of changes in nesting phenology at Playa Grande, where the median nesting date shifted 0.3 days yr^-1^ later between 1993 and 2013 [42]. These changes in phenology are in contrast to studies postulating that increasing ocean temperatures could cause earlier nesting, exposing nests to more favorable conditions [28,43]. 90% poaching of eggs between the mid-1970’s and early 1990’s resulted in a lost generation of nesting females [44]. Beach protection began in the 1990’s and as these nests hatched and females reached reproductive maturity, the nesting population has shifted to a larger proportion of younger and inexperienced females, which tend to nest later in the season [42]. As the nesting season progresses, temperature increases while precipitation decreases, exposing nests to less favorable incubation conditions, which results in lower hatching and emergence success [8]. Later nesting in the season at Playa Grande will place nests in increasingly adverse conditions, likely exacerbating the impacts of climate change. Such shifts make the development of mitigation strategies at the beginning of the nesting season particularly pertinent for this population of leatherbacks.

In addition to increasing reproductive output, watering regimens can also influence hatchling morphology. In loggerhead turtles, eggs incubating in artificial nests with very low gravimetric water content in the sand produced smaller hatchlings, while sand with a gravimetric water content of 0.06 (similar to water content in average and high water treatments in this study) produced the largest hatchlings and also had the highest hatching success [45]. The trend of wetter substrates producing larger hatchlings has been reported for many reptiles, including map turtles (*Graptemys ouachitensis* and *G*. *pseudogeographica*) [46], snapping turtles (*Chelydra Serpentina*) [23], and bull snakes (*Pituophis melanoleucus)* [47]. These larger sizes may incur fitness benefits, as larger turtle hatchlings exhibit greater survivorship [48], greater crawling speeds [49], and better swimming capability. High water treatments in this study corresponded to high levels of rain that resulted in the highest hatching success and emergence of leatherback hatchlings at Playa Grande [8]. Even if watering is less effective at reducing sand temperature, it is still beneficial because it keeps moisture in the sand. Moist sand results in higher hatching success and may produce hatchlings with higher fitness levels while greatly increasing hatchling production.

Watering regimens should be applied with caution, however, as excessive water can be harmful. At greater than 0.06 g g^-1^ gravimetric water content, loggerhead hatchlings become smaller and hatching success decreases [45]. Saturation of the sand with water or flooding of the nest could prevent gas exchange in the nest and reduce hatching success [50]. However, gravimetric water contents of 0.05 to 0.08 g g^-1^ correspond to matric water potentials of about -5 kPa and gravimetric water content down to 0.01 still has a matric water potential of about—10 kPa [50] so it is not dry. The water potential difference between the egg (about -900 kPa) and the sand will cause water to enter the egg and it will gain mass, which is why a sea turtle egg usually becomes turgid soon after deposition by absorbing water from the sand [50]. Rainfall will not flood a beach at sea turtle nest depth as long as the nest is above the water table, but will drain down through the sand due to gravity until it reaches equilibrium with the capillary forces between the sand grains. Water potential in the sand column will usually be between -10 and -25 kPa [51]. Only when there is a dry layer at the surface in which all of the water has evaporated and kPa reaches as high as -100,000 kPa will the water potential cause the eggs to dry out.

That was the case when the sand dried out in our non-watered plots and we could not dig down to get a sand sample at the experimental depth. The deep dry front in these plots suggests that eggs incubating under similar conditions would have dried out significantly, likely resulting in lower hatching success. The mitigation strategies that tackle the problems of climate change most comprehensively will likely be those that combine shade to lower temperatures when they get too high for development and watering hatcheries or the beach before eggs are incubated to prevent the negative consequences associated with dry incubation conditions.

We have compared for the first time the relative impacts of mitigation strategies on different depths in the sand. This is an important consideration in mitigation strategies, as depth interacted significantly with both shade and water. Additionally, water content tended to be higher at 75cm, likely due to evaporation in the top layer of the sand. This trend is supported by analyses of water content on the beach at Playa Grande, which showed that gravimetric water content is significantly higher at 75cm than at 45 cm. Mitigation strategies should therefore be species-specific, as both shade and water treatments will impact the temperature and water content of clutches differently at different sand depths.

Techniques will also be dependent on the objectives of the mitigation strategy. The transitional temperature range for olive ridleys in Costa Rica is around 4°C [52]. Shade treatment without water decreases the temperature 2°C in comparison to controls, but adding water with shade decreases temperature about 4°C in comparison to controls. This means that shading with water may be capable of turning the nest from 100% female to 100% male. In cases where skewed sex ratios present a serious conservation concern [43,53], shifting sex ratios in some nests by combining shade and water may be a desirable outcome in future scenarios of climate change. However, if current sex ratios are not problematic, then shading can be done at times when temperatures do not alter sex ratios to facilitate hatchling survival from the nest and increase reproductive output.

Altering sand temperatures not only changes hatchling sex ratios but may also impact the operational sex ratios of adults [9] and population dynamics [10]. Reconstructing a long-term data set of incubation temperatures is essential for determining mitigation measures because short-term data sets may not encompass variation in hatchling sex ratios that can occur over multiple decades [15]. Thus, while sex ratios may be female-biased over a few years, over longer time spans the operational sex ratio may ultimately approach 1:1. Furthermore, natural shifts in hatchling sex ratios towards more females as temperatures increase may be adaptive, as it increases fecundity [10], although taken to extremes, female biased sex ratios can negatively impact populations. Understanding natural shifts and annual variation in hatchling sex ratios and how these impact population dynamics should be the preliminary steps in developing an appropriate mitigation strategy.

In addition to this information, local environmental conditions will also impact mitigation strategies. Turtles may be able to nest under vegetation on some beaches, reducing the need for shading. On Playa Grande, however, leatherbacks tend to nest on the open beach and there is no vegetation nearby that could offer shade to nests [54]. Shading can have the opposite effect of what we observed if shaded structures prevent rain from reaching nests [38]. Because our study was conducted during the dry season, there was no rainfall and this was therefore not an issue. Additionally, applying water to nests that has been allowed to reach ambient temperatures can result in higher sand temperatures [38]. This was not an outcome in our study because we used tap water. Designing appropriate irrigation techniques is important to ensure that mitigation strategies have the intended effects.

Future studies should test these techniques on actual clutches to examine their impacts on hatching and emergence success, as well as hatchling morphology and sex ratios. While further research is required before the implementation of such techniques, these results provide a foundation for understanding how temperature and water content in sea turtle nests will respond to climate mitigation strategies.
